# Evaluation of clinical parallel workflow in online adaptive MR-guided Radiotherapy: A detailed assessment of treatment session times

**DOI:** 10.1016/j.tipsro.2024.100239

**Published:** 2024-02-13

**Authors:** Claudio Votta, Sara Iacovone, Gabriele Turco, Valerio Carrozzo, Marica Vagni, Aurora Scalia, Giuditta Chiloiro, Guenda Meffe, Matteo Nardini, Giulia Panza, Lorenzo Placidi, Angela Romano, Patrizia Cornacchione, Maria Antonietta Gambacorta, Luca Boldrini

**Affiliations:** a Dipartimento di Diagnostica per Immagini, Radioterapia Oncologica ed Ematologia, Fondazione Policlinico Universitario “A. Gemelli” IRCCS, Roma, Italy; b Università Cattolica del Sacro Cuore, Roma, Italy

**Keywords:** MR-guided radiotherapy, Online adaptive radiotherapy, Clinical parallel workflow, Treatment time analysis

## Abstract

**Introduction:**

Advancements in MRI-guided radiotherapy (MRgRT) enable clinical parallel workflows (CPW) for online adaptive planning (oART), allowing medical physicists (MPs), physicians (MDs), and radiation therapists (RTTs) to perform their tasks simultaneously. This study evaluates the impact of this upgrade on the total treatment time by analyzing each step of the current 0.35T-MRgRT workflow.

**Methods:**

The time process of the workflow steps for 254 treatment fractions in 0.35 MRgRT was examined. Patients have been grouped based on disease site, breathing modality (BM) (BHI or FB), and fractionation (stereotactic body RT [SBRT] or standard fractionated long course [LC]). The time spent for the following workflow steps in Adaptive Treatment (ADP) was analyzed: Patient Setup Time (PSt), MRI Acquisition and Matching (MRt), MR Re-contouring Time (RCt), Re-Planning Time (RPt), Treatment Delivery Time (TDt). Also analyzed was the timing of treatments that followed a Simple workflow (SMP), without the online re-planning (PSt + MRt + TDt.).

**Results:**

The time analysis revealed that the ADP workflow (median: 34 min) is significantly (p < 0.05) longer than the SMP workflow (19 min). The time required for ADP treatments is significantly influenced by TDt, constituting 40 % of the total time. The oART steps (RCt + RPt) took 11 min (median), representing 27 % of the entire procedure. Overall, 79.2 % of oART fractions were completed in less than 45 min, and 30.6 % were completed in less than 30 min.

**Conclusion:**

This preliminary analysis, along with the comparative assessment against existing literature, underscores the potential of CPW to diminish the overall treatment duration in MRgRT-oART. Additionally, it suggests the potential for CPW to promote a more integrated multidisciplinary approach in the execution of oART.

## Highlights

• This study investigates the impact of the clinical parallel workflow on the overall treatment session time in MRI-guided radiotherapy.
• It identifies variations in the time taken for different oART steps across different anatomical sites.
• The study also outlines the tasks performed by radiation therapist technologists (RTTs) within the CPW.

## Introduction

Since the introduction of radiation therapy (RT) in cancer treatment, continuous technological advancements in precision of delivery have significantly improved patient outcomes [1]. Image-guided radiotherapy (IGRT) has been a key development in RT and Magnetic Resonance guided-RT (MRgRT) has taken its capabilities a step further by allowing the acquisition of high-quality imaging with onboard 0.35–1.5 T MRI at any time during the treatment workflow [2]. Over the years, this groundbreaking treatment option has evolved, and improved clinical applications continue to emerge [3].

At the time of writing of this article, there are two commercially available MRgRT technologies: Unity (Elekta, Stockholm, Sweden) uses a 1.5 T MRI scanner with a 7 MV Flattening Filter Free (FFF) Linac, while MRIdian (ViewRay, Cleveland, Ohio) joins 0.35 T MRI scanner with 6 MV FFF Linac for radiation delivery [4], [5].

MRgRT has brought into clinical practice the possibility to perform online adaptive radiotherapy (oART) based on the anatomical changes of the day [6], [7]. Online ART allows therapy volumes to be re-contoured every day, while the patient is still on the couch. The dose distribution is then quickly re-optimized to compensate anatomical variations that may have occurred. An optimized plan is then delivered according to the most convenient optimization solution.

Another significant advantage of this technology is real-time tracking of the therapy volumes during the entire RT delivery [8], [9]. This is achieved using MRI images, acquired in cine mode, and automatic gating, a technique that pauses radiation when the target volume moves outside the foreseen treatment area, preventing unnecessary exposure to Organs At Risk (OARs) [10]. However, automatic gating can result in an increase in beam delivery time particularly when inspiratory breath-hold (BHI) is chosen, with respiratory-gated irradiation potentially taking two to five times longer than free breathing (FB) ones [11].

These technical innovations enable the safe delivery of high doses to the target while minimizing the dose to the OARs and pave the way towards new RT indications and dose escalation protocols [12]. Despite the evident benefits, the additional steps required for oART and the reduced gating efficiency may lead to an overall increase in treatment time, posing a challenge for day-to-day clinical implementation and time-machine burden[13], [14]. This means that the implementation of this technology also requires economic sustainability, which is closely linked to the daily treatment volume and, consequently, to the duration of each treatment session.

In this regard, technological developments in the field of MRgRT are focusing not only on improving system's capabilities but also on reducing the overall treatment time. A new version of the MRIdian treatment delivery system (TDS), named A3i was recently approved for clinical use (510 K approval December 2021) [15]. The A3i system includes various software improvements, such as upgraded TDS an integrated patient feedback display (PFD), automatic contouring tools, multiplanar tracking (MPT), Brain TX package and clinical parallel workflow (CPW).

The implementation of CPW enables radiation oncologists (ROs), medical physicists (MPs), and radiation therapists technologists (RTTs) to divide their tasks and work simultaneously while the patients is still on treatment couch. This feature facilitates the parallel execution of all the necessary oART steps that otherwise would typically be performed sequentially.

The primary aim of this study was to assess the impact of the new technology upgrade on the overall treatment time in Online Adaptive 0.35 MRgRT by analyzing each step of the current Adaptive workflow. Additionally, our goal was to report a comprehensive time analysis for each specific workflow type and treatment site.

## Methods

### Study design and patients characteristics

A representative random sample of consecutive patients treated in our institution with the software version 3.0 (V3) (first upgrade of the new A3I software), MRIdian MR-Linac was considered for this analysis. The study was conducted as a prospective analysis of treatment time data collected over a period of a 2-months after the V3 upgrade implementation (February 2023). Patients have been grouped based on disease site, breathing modality (BM) (BHI or FB), and fractionation (stereotactic body RT [SBRT] or standard fractionated long course [LC]). Each patient signed a specific informed consent for therapy delivery and use of data for research purposes.

### MRgRT workflow

The MRgRT workflow typically encompasses several stages: MR simulation, CT simulation, MR contouring, treatment planning, and Daily Treatment Delivery Sessions (DTDS).

The objective of this analysis was to evaluate the Total Room Occupancy Time (TROt) of the DTDS, defined as the duration from the patient's entry into the treatment room to their exit.

The DTDS is managed by the TDS, a component of the MRIdian A3i system, offering CPW for executing treatment plans under MR image guidance.

It enables online plan adaptation, RT Delivery and the acquisition of initial MR planning images required for the planning process.

The TDS comprises an updated Operator Console computer (Apple 27-inch iMac) and two additional Therapy Portals (Apple 16-inch MacBook Pro), expanding clinician capabilities for parallel and remote oART, including plan review and approval, without the need to be physically present in the treatment control room.

The TDS primarily operates in two distinct workflows: Adaptive Treatment (ADP), which optimizes treatment plans based on daily anatomy; and Simple Treatment (SMP), following a conventional radiotherapy process involving MR image-based shifts and treatment without oART.

The selection of patients to receive either the SMP or ADP workflow was based on the location of the disease site and the fractionated schedule. For all SBRT treatments of lesions in close proximity to OARs, an ADP workflow was followed (e.g., pancreas, prostate, liver). In addition, the ADP workflow was used for all patients on the first day of therapy. This approach allowed for the assessment of the treatment's criticality and the determination of the workflow type for subsequent days.

Both workflows are carried out in parallel mode (CPW) by a multidisciplinary team consisting of an RTT, an MD, and an MP, each with a minimum of three years’ experience in MRgRT. The steps of the DTDS for the ADP workflow are detailed below.

For the SMP workflow, all the aspects discussed for ADP apply, except for re-contouring and re-planning considerations.

#### Patients set-up

The simulation and DTDS were carried out with the patient in a headfirst supine position, employing headphones for noise reduction, mirrored glasses, and a bulb alarm.

The patient was positioned using a MR-compatible positioning board (Fluxboard®, Macromedics, Waddinxveen, The Netherlands), which included foot, knee and arm support, as well as personalized pillows to improve patient comfort [16]. Flexible torso coils were positioned according to the targeted areas for scanning during the procedure.

#### MRI acquisition and matching

Daily planning scans, chosen within the imaging workflow and integrated into the treatment plan, are automatically acquired following patient setup and closure of the RF door. These T2/T1-weighted MR scans are acquired using true fast imaging with steady-state precession (3D-TrueFISP) sequence, with acquisition times ranging from 15 to 200 s and an image resolution varying from 1.5 mm to 3 mm, chosen based on the patient's breathing status. An initial alignment between the daily MR and the reference MR is automatically carried out by the TDS. Subsequent adjustments, if needed, are made by RTT under the supervision of the MD. Then, the MD approved the MR-matching, which also served as the image registration for the subsequent contouring phase.

#### oART re-contouring

The oART recontouring allows for the daily update of all the therapy volumes, successfully managing the interfraction variability.

The Gross Tumor Volume (GTV) generally underwent a rigid registration and was not modified unless for macroscopic modifications, while the majority of OARs were deformably registered to the daily MR anatomy, which is generally prone to much greater variability. Adjustments to the GTV or Clinical Target Volume (CTV) were therefore exclusively managed by a MD when deemed needed.

Notably, owing to significant the inter-fractional variability typical of the digestive system, OARss such as the small and large bowel, duodenum, and stomach were rigidly registered and subsequently recontoured.

The RTT reviewed the contouring from the deformable auto-contouring tool and made necessary adjustments. Each RTT has undergone specialized training in contouring through an internal program led by a MD, and possesses practical experience in offline OARs contouring.

In the case of specific anatomical sites (i.e. upper abdomen or thorax), recontouring was restricted within a 3 cm range from the PTV, following the SMART approach [17]. Air bubble contouring and electron density verification tasks were handled by the attending MP [18].

Additionally, geometric structures were generated using auto-delineation rules for adaptive planning purposes. At the end of the process, the MD reviewed all OARs contours, including those generated with deformable registration and adjusted by the RTT, and approved the entire contouring phase with their credentials.

#### oART Re-Planning

The high number of intensity-modulated radiation therapy (IMRT) fields, ranging between 15 and 29 (median 22), and the relatively fewer multileaf collimator (MLC) segment numbers, varying between 34 and 119 (median 69), were employed to achieve a VMAT-like dose distribution [19], [20]. The automatically generated plans (AGPs) of the MRIdian TDS were assessed in terms of target coverage and adherence to the dose constraints for the OARs. The AGPs include the (1) predicted plan, which project the fluences calculated in the original plan onto patient’s anatomy of the day; (2) the fast reoptimized plan, and (3) the fully reoptimized plan. If none of these three generates a satisfying dose distribution, MP proceed to modify the optimization parameters (importance weights, IMRT efficiency and dose normalization). All generated plans were evaluated collaboratively by the MD and the MP, and subsequently, the MD accepted and approved the plan deemed most optimal. The accepted reoptimized plan was evaluated by the MP using an online QA tool employing a second, independent Monte Carlo engine [21].

#### Treatment delivery and real-time tracking

Following oART planning, parameters for real-target gating were verified. Optimal 2D-planes for tracking target volume were identified on volumetric MR setup images. Monitoring on up to three 2D MR-planes, including sagittal, coronal, and axial views, was possible during treatment. Technical parameters for 2D-TrueFISP-MR were determined, including slice number (2–8 frames/second), Field of View (FOV), and spatial resolution (1.5 mm to 3 mm).

Automatic gating was activated and adjustments were made to what is known as the “real target settings”, comprising the GTV as the target structure, PTV as the boundary (3 mm for BHI and 5 mm for FB), and a percentage of ROI varying between 5 and 15 %, according to team clinical judgement [9], [22]. All FB treatments considered in the study have minimal mobility (<3mm). The contours of the target structure were automatically deformed and projected onto the different cine frames, as was the boundary structure, which was rigidly transferred to the cine MR frames to allow for automatic gating [23], [24].

The radiation beam automatically deactivated if the target structure (%ROI) exceeded a predefined threshold, changing RF and electron pulse phases [25].

During delivery, the PFD positioned on the vault's wall behind the bore is activated, displaying the GTV and boundary contours to the patient. When the target aligns within the set boundary for each plane, a smiley face emoji appears, signaling to the patient to maintain the target within the boundary. If the target doesn't align with the set boundary, no emoji appears [26].

### Treatment timeline analysis

The time spent on each step was recorded prospectively for each DTDS using a stopwatch integrated in the TPS.

**Fig. 1:**
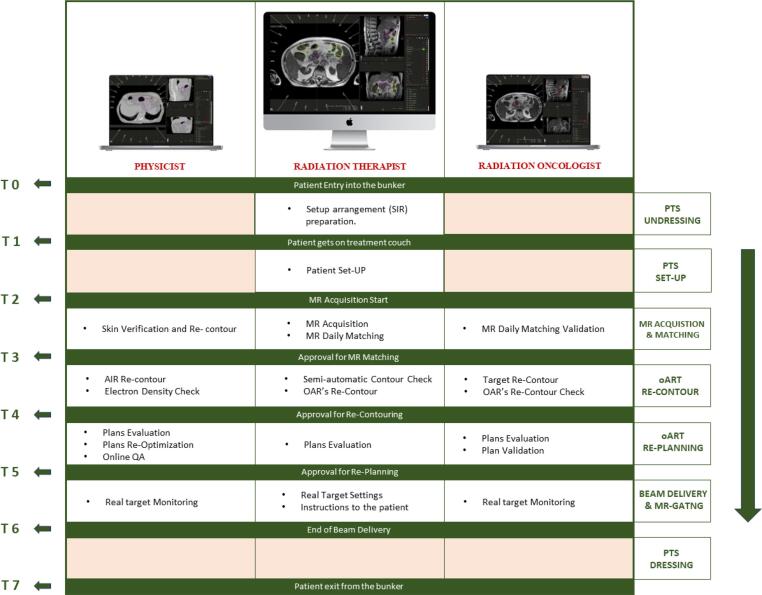
A schematic representation of the various tasks performed by each figure involved in the CPW (MD, RTT and MP) is shown. On the left are the seven moments in which time was recorded; these moments represent the transition from one step to another.

Timekeeping began when the treatment plan is opened, which we synchronized with the patient's entry into the bunker (T0). Other six times (hh:mm:ss) were prospectively documented, and are reported in Fig. 1.

The time spent for the following workflow steps was also analyzed: Patient Entry Time (PEt), Patient Setup Time (PSt), MRI Acquisition and Matching (MRt), MR Re-contouring Time (RCt), Re-Planning Time (RPt), Treatment Delivery Time (TDt), and Patient Exit Time (PEXt).

Total session time (TSt) was assessed by subtracting from the total room occupancy time (TROt) the time each patient spent dressing and undressing (PEt + PEXt), as this variable may differ among centers based on their room architecture and availability of dedicated changing rooms.

Fig. 1 displays a schematic visualization of the various analyzed steps, encompassing the diverse tasks involved in the CPW.

### Statistics

All data collection was performed using Microsoft® Excel (Version 2212 Build 16.0.15928.20196). Subsequent statistical analyses were carried out using Python v 3.9 scripts.

Statistical analyses included the Shapiro-Wilk test to check for normal distribution, independent *t*-test or non-parametric Mann-Whitney *U* test as appropriate. The statistical significance level was set at 0.05.

## Results

### Patient characteristics

The treatment times of 56 patients (27 males and 29 females) were analyzed. Treatment times of 254 fractions were collected for these patients, with 180 (70.9 %) following an ADP workflow and 74 (29.1 %) following a SMP workflow.

Table 1 shows treatment details divided for patients (PTS) and fraction (FX) characteristics.

**Table 1:** Treatment details categorized by patient (PTS) and fraction (FX) characteristics.

			**N**	**[%]**	**Median**	**Range**
**Patients**			**56**			
	**General Info**	Male	27	48,2%		
		Female	29	51,8%		
		Age			66	35–82
	**Plan Info**	SBRT	47	83,9%		
		Standard Fractionation	9	16,1%		
		N FX/day			5	5–28
		Total px dose (Gy)			40	25–55

	**Target Location**					
		Nodes	12	21,4%		
		Rectum	12	21,4%		
		Lung	11	19,6%		
		Pancreas	8	14,3%		
		Liver	7	12,5%		
		Other	3	5,4%		
		Adrenal	2	3,6%		
		Prostate	1	1,8%		

**Fractions**			**254**			
	**Plan info**	Adaptive	180	70,9%		
		Simple	74	29,1%		
		Breath hold	112	44,1%		
		Free Breathing	142	55,9%		
		SBRT	186	73,2%		
		Standard Fractionation	68	26,8%		

	**Target Location**					
		Rectum	68	26,8%		
		Lung	54	21,3%		
		Nodes	52	20,5%		
		Pancreas	29	11,4%		
		Liver	27	10,6%		
		Other	15	5,9%		
		Adrenal	6	2,4%		
		Prostate	3	1,2%		

The primary reasons for adopting an ADP workflow were fractionation (82.7 % of ADP fractions were for SBRT) and site (13.9 % of ADP fractions involved rectum for GTV contouring management). Only 3.3 % of ADP fractions did not exhibit these characteristics.

Overall, BHI delivery was utilized in 112 fractions (44.1 % out of 256). FB with gating was employed in 142 fractions (55.9 %), predominantly among patients with pelvic lesions or lesions not influenced by respiratory motion.

The most frequently analyzed treatment sites were the rectum (26.8 %), lung (21.3 %), and nodes (20.5 %). Pancreas accounted for 16.1 % of fractions following an ADP workflow. Additionally, two paravertebral lesions and a mediastinal nodule were categorized as “other” (5.4 %).

### Time analysis of MRgRT-CPW

The time analysis for the ADP and SMP workflow components is detailed in Table 2 of the supplementary materials.

**Fig. 2:**
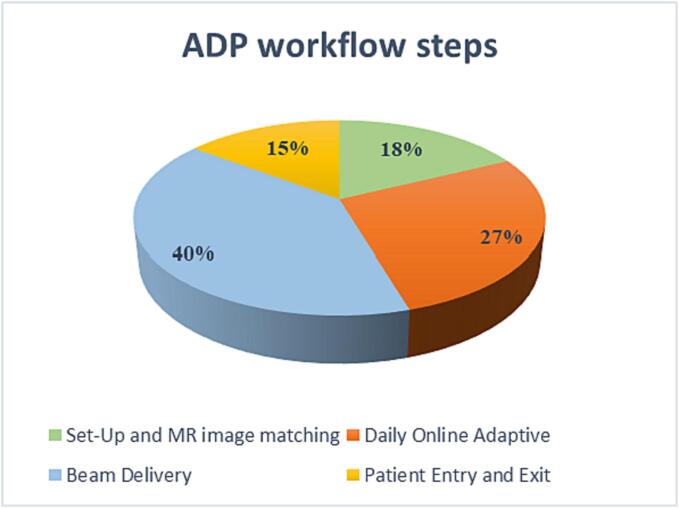
Pie chart showing the percentage of time spent on each macro-step of the ADP workflow.

The time analysis (h:mm) showed that the ADP workflow (median: 0:34, range: 0:21–1:40) is significantly (p < 0.05) longer than the SMP workflow (median: 0:19, range: 0:11–0:38). This is attributed to the fact that plan adaptation represents 27 % of the entire procedure (as shown in Fig. 2) and that the majority of ADP treatments (82.8 %) involves SBRT.

Furthermore, there is a statistically significant difference (SSD) (p < 0.05) between the oART cases in which a re-optimisation of the plan is required (median TSt: 0:36) and the cases in which the predictive plan is delivered (median TSt: 0:28).

**Fig. 3:**
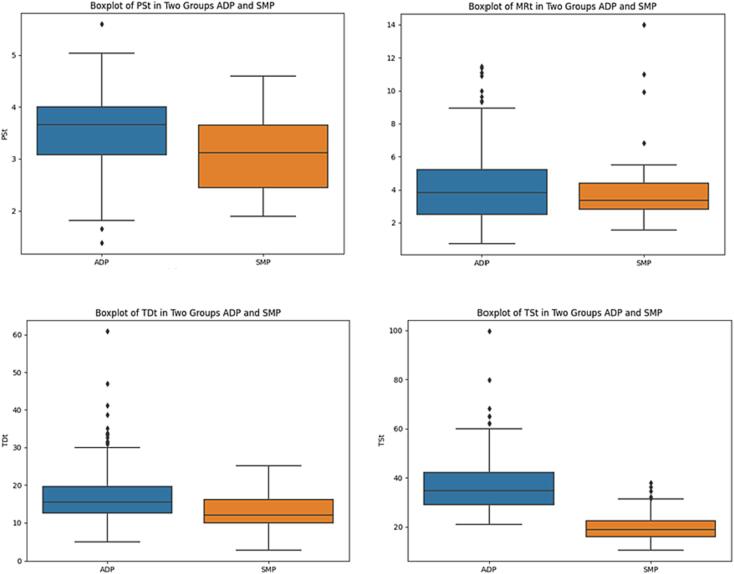
Boxplots of common Step Duration (min) in Two Groups: ADP and SMP.

Fig. 3 illustrates the differences in various common steps between ADP and SMP treatment workflows.

There is a SSD in both patient setup and beam delivery between these two workflows.

This difference is likely attributed to the fact that half of the SMP cases (49 %) involves pelvic treatments, resulting in faster set-up and delivery in FB conditions.

The time required for oART treatment is significantly influenced by beam delivery, constituting 40 % of the total time. In this context, the breathing modality represents a crucial factor determining the overall duration of the treatment: indeed, a SSD (p < 0.05) emerges between the BHI treatments for both ADP workflow (median BHI: 0:38; median FB: 0:31) and SMP workflow (median BHI: 0:23; median FB: 0:18).

No SSD is observed between SBRT treatments (median TSt: 0:35) and LC treatments (median TSt: 0:35) in the ADP workflow. However, a SSD is evident between these two categories in the SMP workflow (median SBRT-TSt: 0:22; median LC-TSt: 0:17). This is attributed to the fact that 80 % of ADP-LC treatments involves the rectum, necessitating an oART (RCt + RPt) time of 0:16 (median), making it the most time-consuming anatomical site regarding oART steps.

The anatomical site requiring a longer total time is the lung (median TSt: 0:40), likely due to an extended beam delivery time (median TDt: 0:21), influenced by BHI status.

**Fig. 4:**
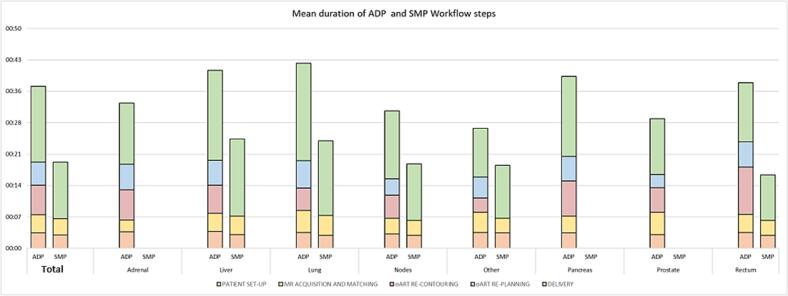
Bar chart of mean duration of ADP and SMP workflow steps for different target locations.

Fig. 4 shows a detailed analysis of the timing of various steps per anatomical site.

Overall, 79.2 % of oART fractions were completed in less than 45 min, and the 30.6 % was completed in less than 30 min.

## Discussion

In this study, the workflow timing of a 0.35 T MRgRT treatment was analyzed. Both the ADP workflow and the SMP treatment duration, which follows a conventional workflow, were examined.

It has been reported in the literature that MRgRT treatments are significantly longer than conventional RT treatments [13], [27], [28], [29]. This aspect constituted one of the major obstacles to the large-scale implementation of these systems since its introduction in clinical practice [30].

In this context, scientific advancements in this field are progressing to reduce the overall treatment time, either by implementing artificial intelligence (AI) systems, such as auto contouring and auto planning, or by introducing software that enables the simultaneous execution of different tasks required during oART-MRgRT such as the CPW [31], [32], [33], [34].

In the past, various studies have developed different techniques to reduce this timeframe. Bohoudi et al. proposed a rapid oART approach for pancreatic cancer in SBRT with 0.35 T MRgRT, performing the re-contouring of the OARs within 3 cm of the target only [17]. This strategy resulted in a mean oART timeframe of 12 ± 4 min (min). For similar anatomical sites (upper abdomen), Güngör et al. highlighted a mean duration for these steps of 17.8 ± 3.9 min with standard sequential execution [13]. In our first month of CPW experience, the mean time for these steps in the upper abdomen oART was 13 min (mean RCt = 7,3 min and mean RPt = 5,6 min), meaning that we have achieved a reduction in oART steps time of around 30 %.

Tyagi et al. reported a median contouring time of 23 min and a median planning time of 10 min for oART pancreas SBRT treatments on the 1.5 T system, while Uno et al. reported a median time for pancreatic oART steps (contouring and planning) of 46 min[35], [36].

Lamb et al. estimated a median execution fraction time of 54 min on 80 cases, with contouring identified as the most time-consuming step (mean time: 22 min) [37]. For pancreatic oART, Tyagi et al. reported a median total treatment time of 75 (49–152 min) using a 1.5 T MR Linac [35].

In our CPW-based experience, pancreatic oART had a median duration of 39 (25–59) min.

In brief, our analysis revealed that treatment sessions following an ADP workflow had a median duration of 34 min (range: 21–100 min), while sessions following a SMP workflow were 19 min (range: 11–38 min). With the previous version of the 0.35 T MR Linac system, which employs a sequential workflow, Gungor's time analysis showed a median total treatment time of 45 min in the ADP workflow [13]. Since these results are fully consistent with our institutional timelines prior to the implementation of the CPW, we believe that the CPW has reduced the time for oART fractions by about 25 %.

Our study documented that among all the steps of the adaptive workflow, delivery was the most time-consuming one, accounting for 40 % of the TROt, with a median duration of 15 min. Not surprisingly, the utilization of BHI treatment plans led to a notable increase in beam delivery time compared to free FB. The efficiency of BHI treatments was closely associated with robust patient cooperation and the consistent reproducibility of the breathing pattern. To further improve this, an integrated audiovisual coaching system incorporating a prismatic glass mirror and a TDS screen positioned at the patient's back, was successfully employed [26], [38].

The longer duration of MRgRT treatment can be explained not only with the possibility to re-optimize the dose on the anatomy of the day, but also with the fact that the treatments that benefit the most from this technology, often foresee high dose per fraction and low gating efficiency, due to the many moments when the beam is off due to direct gating of the tumor.

Analyzing standard fractionated MRgRT treatments (without ADP, SBRT, and BHI), we observed a total median session duration of 17 min (range: 11–21), which is comparable to conventional RT treatments [39], [40].

However, a parallel workflow necessitates close multidisciplinary collaboration involving MDs, MPs and RTTs. The latter are actively engaged in managing the Control Workstation that oversees the entire workflow and may act as pacemakers of the entire process.

The RTTs' ability to compare MR images, segment OARs and evaluate the treatment plan with MPs and MDs enables the synchronous utilization of the three workstations administering the oART process, resulting in a reduction in treatment time [41].

Various training strategies were developed to facilitate a therapist-driven workflow for oART on the MR-Linac [42]. Li et al. proposed a practice-based strategy for RTTs in the management of prostate oART treatments; this strategy is based on three phases: contouring phase, online assessment, and ongoing quality control [43].

This necessitates the creation of an enhanced RTT practice model, encompassing the acquisition of MRgART-specific skills, knowledge and competencies required to optimize the service and harness new innovations in this field [44], [45].

Despite being the first evaluation of the impact of CPW on MRgRT delivery, this study has some limitations. Firstly, the analysis considered a limited number of fractions only, potentially omitting variables that could influence treatment times.

Secondly, the data only reflects the initial period of implementation of the new A3i software, which may not accurately represent its long-term impact on treatment efficiency, also due the learning curve effect. Further studies and a longer follow-up period would be necessary to assess and determine its true impact on treatment times. Despite these limitations, this preliminary analysis and the comparison with the literature highlight how the CPW could reduce the occupancy time in MRgRT oART treatments. In our clinical practice, this data has already facilitated an increase not only in the number of daily treated patients but also in the percentage of fractions following an ADP workflow (from 20 % to 60 %). In subsequent research, a systematic review could rigorously explore variations in oART times, taking into account the diverse technological and methodological approaches utilized. Such an inquiry would facilitate a more accurate quantification of the specific impact attributable to the CPW. However, this type of comparison should take into account not only the MRgRT system used but also the treatment protocols, which vary from center to center.

## Conclusion

MRgRT implementation may be challenging due to prolonged treatment times, limiting accessibility for certain cancer types. To the best of our knowledge, this is the first study that demonstrated the functionalities of CPW and analyzes the positive impact of this technology on total MRgRT treatment time. Looking ahead, enhanced AI integration and increased experience with adaptive CPW could further shorten oART treatments, making this technology more widely adopted and improving accessibility for all patients.

## CRediT authorship contribution statement

**Claudio Votta:** Conceptualization, Data curation, Investigation, Methodology, Writing – original draft, Formal analysis, Project administration. **Sara Iacovone:** Data curation, Visualization, Review & editing. **Gabriele Turco:** Data curation, Visualization, Review & editing. **Valerio Carrozzo:** Data curation, Visualization, Review & editing. **Marica Vagni:** Statistical Analysis, Data curation, Visualization, Review & editing. **Aurora Scalia:** Data curation, Visualization, Review & editing. **Giuditta Chiloiro:** Validation, Visualization, Review & editing. **Guenda Meffe:** Validation, Visualization, Review & editing. **Matteo Nardini:** Validation, Visualization, Writing – review & editing. **Giulia Panza:** Validation, Visualization, Writing – review & editing. **Lorenzo Placidi:** Validation, Visualization, Review & editing. **Angela Romano:** Validation, Visualization, Review & editing. **Patrizia Cornacchione:** Validation, Visualization, Review & editing. **Maria Antonietta Gambacorta:** Supervision, Validation, Visualization, Review & editing. **Luca Boldrini:** Supervision, Validation, Visualization, Writing – review & editing.

## Declaration of competing interest

The authors declare that they have no known competing financial interests or personal relationships that could have appeared to influence the work reported in this paper.
