# Supplementary material for: Evaluation of clinical parallel workflow in online adaptive MR-guided Radiotherapy: A detailed assessment of treatment session times
Source: Tech Innov Patient Support Radiat Oncol. 2024 Feb 13;29:100239. doi: 10.1016/j.tipsro.2024.100239 (PMC10883837; doi:10.1016/j.tipsro.2024.100239)
Supplement: Supplementary Data 1 [file mmc1.pdf]

Supplementary Materials

Table 2			Median (range) time analysis of ADP and SMO workflow steps according to treatment site, breathing status (BS) and dose fractionation					
			Patient Set-up PSt	MRI Acquisition and Matching MRt	Daily MR Re-contouring RCt	Daily MR Re-Planning RPt	Treatment Delivery TDt	Total Session Time (TSt)
**Adaptive**		Total	0:03:21 (0:01:23 - 0:05:36)	0:03:30 (0:00:44 - 0:11:29)	0:06:00 (0:00:21 - 0:26:20)	0:04:23 (0:00:52 - 0:38:35)	0:14:23 (0:05:10 - 1:00:56)	0:33:39 (0:21:05 - 1:39:44)
	**Site**	Adrenal	0:03:46 (0:03:14 - 0:04:20)	0:02:42 (0:01:11 - 0:04:01)	0:07:39 (0:03:28 - 0:07:46)	0:05:26 (0:03:11 - 0:10:10)	0:13:59 (0:11:01 - 0:17:22)	0:33:01 (0:30:29 - 0:36:08)
		Liver	0:03:57 (0:02:40 - 0:05:36)	0:04:37 (0:00:44 - 0:09:38)	0:05:22 (0:01:25 - 0:22:07)	0:04:38 (0:01:20 - 0:14:53)	0:18:27 (0:12:18 - 0:38:40)	0:37:27 (0:26:45 - 1:04:56)
		Lung	0:03:46 (0:02:09 - 0:04:50)	0:04:13 (0:01:10 - 0:11:29)	0:03:33 (0:00:30 - 0:14:03)	0:05:22 (0:00:52 - 0:38:35)	0:20:54 (0:07:00 - 1:00:56)	0:40:13 (0:21:05 - 1:38:44)
		Nodes	0:03:17 (0:01:39 - 0:04:36)	0:03:21 (0:01:00 - 0:08:49)	0:05:15 (0:01:03 - 0:11:41)	0:02:59 (0:00:52 - 0:11:27)	0:13:41 (0:07:00 - 0:33:20)	0:29:19 (0:21:05 - 0:48:23)
		Other	0:03:19 (0:02:49 - 0:04:49)	0:04:33 (0:02:24 - 0:06:51)	0:02:46 (0:01:30 - 0:05:55)	0:04:26 (0:00:58 - 0:09:48)	0:11:08 (0:08:27 - 0:13:22)	0:26:05 (0:22:27 - 0:34:48)
		Pancreas	0:03:40 (0:01:23 - 0:04:55)	0:03:22 (0:01:10 - 0:11:29)	0:07:02 (0:01:50 - 0:26:20)	0:04:55 (0:02:30 - 0:38:35)	0:16:29 (0:12:46 - 0:41:13)	0:38:55 (0:25:16 - 1:08:07)
		Prostate	0:03:01 (0:02:35 - 0:03:47)	0:05:35 (0:02:52 - 0:06:57)	0:04:47 (0:02:50 - 0:09:22)	0:02:25 (0:02:08 - 0:04:18)	0:12:56 (0:12:13 - 0:13:15)	0:28:02 (0:25:01 - 0:35:58)
		Rectum	0:03:39 (0:01:23 - 0:05:02)	0:04:02 (0:00:44 - 0:11:29)	00:10:37 (0:00:21 - 0:26:20)	0:05:00 (0:00:57 - 0:38:35)	0:13:15 (0:05:10 - 1:00:56)	0:37:21 (00:21:05 - 1:39:44)
	**BS**	BHI	0:03:46 (0:01:23 - 0:05:36)	0:03:49 (0:00:44 - 0:11:29)	0:06:02 (0:00:21 - 0:26:20)	0:04:38 (0:00:52 - 0:38:35)	0:18:38 (0:05:10 - 1:00:56)	0:38:02 (0:21:05 - 1:39:44)
		FB	0:03:26 (0:01:39 - 0:04:55)	0:03:49 (0:00:50 - 0:10:55)	0:05:58 (0:00:21 - 0:26:20)	0:04:10 (0:00:57 - 0:15:50)	0:12:39 (0:05:10 - 0:33:20)	0:31:09 (0:21:05 - 0:54:32)
	**Dose x Day**	SBRT	0:03:43 (0:01:23 - 0:05:36)	0:03:40 (0:00:44 - 0:11:29)	0:05:36 (0:00:30 - 0:25:40)	0:04:07 (0:00:52 - 0:38:35)	0:15.50 (0:05:10 - 1:00:56)	0:33:44 (0:21:05 - 1:39:44)
		LC	0:03:36 (0:02:11 - 0:04:36)	0:04:00 (0:01:47 - 0:09:59)	0:09:52 (0:0:21 - 0:26:20)	0:05:00 (0:02:30 - 0:12:57)	0:13:05 (0:05:40 - 0:18:29)	0:36:06 (0:21:05 - 0:52:31)
**Simple**		Total	0:03:07 (0:01:54 - 0:04:36)	0:03:22 (0:01:33 - 0:14:00)	N/A	N/A	0:11:59 (0:02:54 - 0:25:10)	0:18:48 (0:10:51 - 0:38:02)
	**Site**	Adrenal	N/A	N/A	N/A	N/A	N/A	N/A
		Liver	0:03:01 (0:02:35 - 0:03:52)	0:03:56 (0:02:37 - 0:06:50)	N/A	N/A	0:17:37 (0:11:38 - 0:24:34)	0:24:45 (0:17:09 - 0:31:32)
		Lung	0:03:04 (0:01:54 - 0:04:20)	0:03:26 (0:02:50 - 0:11:00)	N/A	N/A	0:17:00 (0:10:00 - 0:25:10)	0:23:26 (0:16:36 - 0:38:02)
		Nodes	0:02:54 (0:01:57 - 0:03:58)	0:03:16 (0:01:33 - 0:05:30)	N/A	N/A	0:13:42 (0:05:00 - 0:18:57)	0:20:57 (0:13:51 - 0:23:01)
		Other	0:03:40 (0:02:38 - 0:04:36)	0:03:45 (0:01:40 - 0:04:41)	N/A	N/A	0:12:12 (0:11:00 - 0:12:41)	0:18:36 (0:16:40 - 0:21:36)
		Pancreas	N/A	N/A N/A	N/A	N/A	N/A	N/A
		Prostate	N/A	N/A N/A	N/A	N/A	N/A	N/A
		Rectum	0:03:10 (0:01:57 - 0:03:59)	0:03:14 (0:01:50 - 0:14:00)	N/A	N/A	0:10:17 (0:02:54 - 0:16:10)	0:16:51 (0:10:51 - 0:21:04)
	**BS**	BHI	0:02:57 (0:01:54 - 0:03:52)	0:03:56 (0:02:37 - 0:09:55)	N/A	N/A	0:13:56 (0:10:02 - 0:24:34)	0:22:42 (0:16:39 - 0:36:10)
		FB	0:03:22 (0:01:57 - 0:04:36)	0:03:22 (0:01:33 - 0:14:00)	N/A	N/A	0:11:00 (0:02:54 - 0:25:10)	0:18:14 (0:10:51 - 0:38:02)
	**Dose x Day**	SBRT	0:03:01 (0:01:54 - 0:04:36)	0:03:45 (0:01:33 - 0:11.00)	N/A	N/A	0:15:38 (0:05:00 - 0:25:10)	0:22:27 (0:13:51 - 0:38:02)
		LC	0:03:10 (0:01:57 - 0:03:59)	0:03:13 (0:01:50 - 0:14:00)	N/A	N/A	0:10:16 (0:02:54 - 0:16:10)	0:16:50 (0:10:51 - 0:21:04)
